# Size-based characterization of adalimumab and TNF-α interactions using flow induced dispersion analysis: assessment of avidity-stabilized multiple bound species

**DOI:** 10.1038/s41598-021-84113-z

**Published:** 2021-02-26

**Authors:** Morten E. Pedersen, Ragna M. S. Haegebaert, Jesper Østergaard, Henrik Jensen

**Affiliations:** 1 Fida Biosystems ApS, Fruebjergvej 3, 2100 Copenhagen O, Denmark; 2 Department of Pharmacy, University of Copenhagen, Universitetsparken 2, 2100 Copenhagen O, Denmark

**Keywords:** Biochemistry, Biophysics, Drug discovery, Chemistry

## Abstract

The understanding and characterization of protein interactions is crucial for elucidation of complicated biomolecular processes as well as for the development of new biopharmaceutical therapies. Often, protein interactions involve multiple binding, avidity, oligomerization, and are dependent on the local environment. Current analytical methodologies are unable to provide a detailed mechanistic characterization considering all these parameters, since they often rely on surface immobilization, cannot measure under biorelevant conditions, or do not feature a structurally-related readout for indicating formation of multiple bound species. In this work, we report the use of flow induced dispersion analysis (FIDA) for in-solution characterization of complex protein interactions under in vivo like conditions. FIDA is an immobilization-free ligand binding methodology employing Taylor dispersion analysis for measuring the hydrodynamic radius (size) of biomolecular complexes. Here, the FIDA technology is utilized for a size-based characterization of the interaction between TNF-α and adalimumab. We report concentration-dependent complex sizes, binding affinities (*K*_d_), kinetics, and higher order stoichiometries, thus providing essential information on the TNF-α–adalimumab binding mechanism. Furthermore, it is shown that the avidity stabilized complexes involving formation of multiple non-covalent bonds are formed on a longer timescale than the primary complexes formed in a simple 1 to 1 binding event.

## Introduction

The elucidation of biomolecular interactions is key to understanding physiological processes on a molecular level as well as for developing novel pharmaceutical therapies^1–5^. Hence, appropriate analytical methodologies are required in order to comprehensively characterize and quantify these interactions^4–7^. This is often achieved via functional assays such as ligand binding assays (LBA) and cell-based assays, that serve as an important surrogate of the anticipated biological mechanism in patients^4–7^. Ligand binding technologies are capable of reporting apparent binding affinities, but most methods lack a detailed deconvolution on the structural mode of action^6,8^. Often the relevant interactions involve higher order stoichiometries driven by avidity and oligomerization; rarely a simple 1:1 binding model provides a full picture of the underlying biology^3,9,10^. For example, the oligomeric state of a protein may determine whether it is pathological or not, as seen with tumor necrosis alpha (TNF-α) where the TNF-α monomer and trimer are inactive and active inflammatory cytokines, respectively^11^. It is therefore often necessary to combine LBAs with structural methods (e.g. size-exclusion chromatography, SDS-PAGE, mass spectrometry) to get the full picture^4–7^. However, structural methodologies require relatively clean samples, which may not reflect true *in-vivo* conditions^12^. Ideally, functional assays should report an absolute measure of affinity in combination with structural information under biorelevant conditions.

Flow Induced Dispersion Analysis (FIDA) is a novel analytical platform for size-based characterization of biomolecules including assessment of binding affinity (*K*_d_), complex hydrodynamic radius, analyte quantification, and physical stability^13–17^. The FIDA principle relies on Taylor dispersion analysis (TDA) for measuring the apparent hydrodynamic radius (size) of a selective ligand (the indicator) as it binds to the analyte (e.g., the protein/biomolecule) of interest^13^. The apparent size of the indicator is measured in a titration series with varying analyte concentrations. The resulting binding curve is used for determining the binding affinity (*K*_d_) and complex size. FIDA is highly tolerant to analysis in complex and crude sample matrices, such as plasma and fermentation broth^15,17,18^, and the absolute size readout provides information on structural features of the formed complex as well as avidity stabilization of multiple bound species.

Adalimumab is a recombinant monoclonal IgG antibody, frequently used in the treatment of auto-immune diseases such as rheumatoid arthritis and Crohn’s disease^9,19^. It binds and neutralizes the inflammatory cytokine TNF-α with high affinity and specificity^9,19^. The interaction between TNF-α and adalimumab has been characterized applying various techniques such as surface plasmon resonance (SPR), bio-layer interferometry (BLI), isothermal titration calorimetry (ITC), electron microscopy (EM), dynamic light scattering (DLS), analytical ultracentrifugation and HPLC-based procedures^10,20–24^. However, under physiological conditions TNF-α is a trimer with up to 3 binding sites and adalimumab has two binding sites which may make characterization of the TNF-α–adalimumab interaction challenging. In particular, surface-based methodologies lack the possibility of easily controlling the concentrations of both binding partners, which complicates quantitative assessment of avidity effects. For instance, the TNF-α–adalimumab interaction was recently characterized using hydrodynamic friction measurements^25^, which confirmed strong avidity effects and the presence of higher order complexes. Most of the existing studies of the TNF-α–adalimumab system have been primarily conducted in neat buffer solutions, hence, little is known on TNF-α–adalimumab interactions under native (in-solution) and biorelevant conditions (e.g., in human plasma). *Krayukhina *et al*.* recently utilized analytical ultracentrifugation (AUC) employing fluorescence detection to assess different binding stoichiometries of TNF-α and adalimumab in buffer and human serum^22^. While AUC remains an important methodology, throughput and sample volume requirements limits its broad applicability.

In this work, we have developed FIDA assays for size-based characterization of the interaction between trimeric TNF-α and adalimumab in buffer as well as human plasma. The assays were based on TNF-α as the indicator (ligand); hence, the apparent size change of TNF-α was measured in the presence of varying adalimumab concentrations. We report the in-solution binding affinity (*K*_d_) and avidity stabilized complexes, size of the formed complexes, stoichiometry modeling, and qualitative assessment of the kinetics involved in formation of lower and higher order complexes.

## Results and discussion

### Characterization of TNF-α and adalimumab interactions in pre-incubated buffer samples

Characterization of biomolecular interactions using FIDA is based on an indicator molecule, TNF-α, selectively interacting with the analyte of interest, adalimumab. Initially, the hydrodynamic radius (size) of TNF-α is measured in the absence of adalimumab, thus the size of unbound TNF-α is measured confirming the structural integrity of the molecule. As the concentration of adalimumab is increased, the apparent size of TNF-α increases due to a higher degree of binding. Full details on how to attain the hydrodynamic radius of the indicator from the raw data, i.e. Taylorgrams, has been described elsewhere^14,17^. The apparent TNF-α size is plotted as a function of adalimumab concentration, thereby providing a means for in-solution characterization of affinity (*K*_d_), stoichiometry, and complex size.

The hydrodynamic radii of unbound TNF-α-alexa488 (100 nM) and adalimumab (1 mg/mL) were determined by TDA to 3.20 ± 0.04 nm and 5.6 ± 0.24 nm, respectively. The size of TNF-α confirmed the expected oligomeric state as trimeric at physiological pH 7.4. Kohno et al. reported a hydrodynamic radius of 3.1 nm for the trimer using SEC-LC at the same pH^20^, and Daub et al. found the structure of TNF-α as trimeric at 100 nM (pH 7.4) using hydrodynamic friction measurements^25^. The determined hydrodynamic radius of adalimumab was consistent with previous TDA measurements (5.6–5.9 nm)^26^.

**Figure 1:**
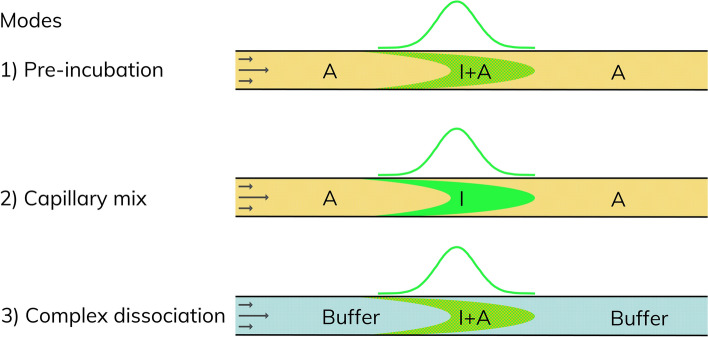
Schematic showing the applied incubation and mixing modes inside the capillary of the FIDA experiments, allowing characterization of in-solution binding kinetics. I, A, and I + A represent solutions of indicator, analyte, and indicator pre-incubated with analyte, respectively.

Three different incubation and mixing modes were used (see Fig. 1): (1) Pre-incubation where TNF-α-alexa488 and adalimumab were pre-incubated (> 10 min) prior to the FIDA measurements, and subsequently analyzed with adjacent zones of equal adalimumab concentration; (2) Capillary mix where TNF-α-alexa488 was mixed with adalimumab inside the capillary during the mobilization period (~ 2–3 min); (3) Complex dissociation where TNF-α-alexa488 and adalimumab were pre-incubated (> 10 min) prior to the FIDA measurements, and then analyzed with adjacent zones of neat assay buffer.

**Figure 2:**
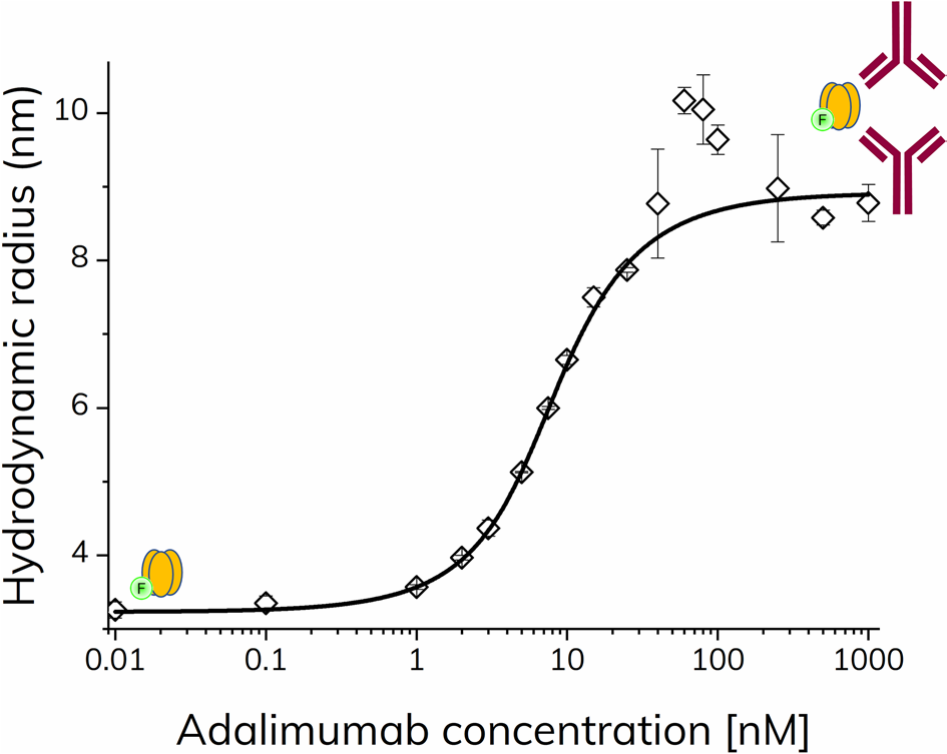
Apparent hydrodynamic radius of TNF-α-alexa488 (100 nM) as function of adalimumab concentration (0 – 1000 nM) determined by FIDA in 67 mM phosphate buffer with 0.1% v/v HSA at pH 7.40 and 25 °C using pre-incubated samples (n = 3, error bars represent standard deviation). The solid line shows fitting to the excess indicator binding isotherm [Eq. (7)].

In the first set of experiments the pre-incubation approach was used. The apparent size of TNF-α-alexa488 (100 nM) was measured throughout an adalimumab titration series (0–1000 nM) in assay buffer containing 0.1% v/v HSA, which was added in order to minimize surface adsorption of TNF-α to glass vials and pipette tips. The apparent size of TNF-α increased with increasing adalimumab concentrations resulting in a binding curve (Fig. 2). The increase in apparent size was due to the formation of TNF-α–adalimumab complexes. At 60–120 nM adalimumab, the binding curve exhibited apparent complex sizes up to 10 nm, which declined to around 8.5 nm with adalimumab concentrations above 120 nM. This led to a hook-shaped binding curve (Fig. 2) indicating that a simple 1:1 binding does not apply to the TNF-α–adalimumab interaction and that larger complexes are formed under certain conditions. Trimeric TNF-α possesses three binding sites and adalimumab is an antibody with two binding sites, hence allowing for formation of various binding stoichiometries. The formation of higher order complexes is favored when the concentrations of TNF-α and adalimumab are nearly similar, as seen in Fig. 2. This is in agreement with previous findings where different complex stoichiometries have been observed^20–23^. The concentrations and formation of higher order complexes are heavily governed by the absolute and relative concentrations of the interacting species. Thus, at excess adalimumab concentrations (500–1000 nM, Fig. 2), the complex formation was shifted towards smaller species. This was also observed in a study using analytical ultracentrifugation and DLS^22^.

The simple 1:1 excess binding model [Eq. (7)] appeared to fit the data well (Fig. 2, solid line), except for concentrations where higher order structures were favored, resulting in an apparent *K*_d_ of 1.52 nM and complex size of 8.9 nm (R^2^: 0.992).

Collectively, these results confirmed the oligomeric state of TNF-α-alexa488 as trimeric from the size measurement of unbound TNF-α as well as its ability to form higher order complexes with adalimumab. The size measurements and binding isotherm provide some information on the structural features of the complexes being formed, however, the results clearly indicate that a more advanced binding model is needed to further quantify the formation of higher order complexes, as well as taking avidity effects into account. For systems where no prior knowledge on the complex stoichiometry is available, it may be necessary to include orthogonal techniques.

### Extended binding model of TNF-α and adalimumab interactions in pre-incubated buffer samples

A range of different TNF-α–adalimumab stoichiometries, e.g., 1:1, 1:2, 2:1, 2:2, 3:2, has been reported^20–23^. On this basis, we set out to derive an extended binding model to describe the data set in Fig. 2. The binding scheme below is suggested to represent a better description of the TNF-α–adalimumab interactions considering the observed higher order structures:

1 $$ {\text{TA}} \rightleftharpoons {\text{T }} + {\text{ A}}\;\;\;\;\;\;\;K_{{{\text{d}}_{1} }} $$

2 $$ {\text{T}}_{{2}} {\text{A}} \rightleftharpoons {\text{TA }} + {\text{ T}}\;\;\;\;\;\;\;K_{{{\text{d}}_{2} }} $$

3 $$ {\text{TA}}_{{2}} \rightleftharpoons {\text{TA }} + {\text{ A}}\;\;\;\;\;\;\;K_{{{\text{d}}_{3} }} $$

4 $$ \left( {{\text{TA}}} \right)_{{2}} \rightleftharpoons {\text{TA }} + {\text{ TA}}\;\;\;\;\;\;\;K_{{{\text{d}}_{4} }} $$

5 $$ \left( {{\text{TA}}} \right)_{{2}} \rightleftharpoons {\text{T}}_{{2}} {\text{A }} + {\text{ A}}\;\;\;\;\;\;\;K_{{{\text{d}}_{5} }} $$

6 $$ \left( {{\text{TA}}} \right)_{{2}} \rightleftharpoons {\text{TA}}_{{2}} \, + {\text{ T}}\;\;\;\;\;\;\;K_{{{\text{d}}_{6} }} $$

where T and A represent trimeric TNF-α and adalimumab, respectively.

**Figure 3:**
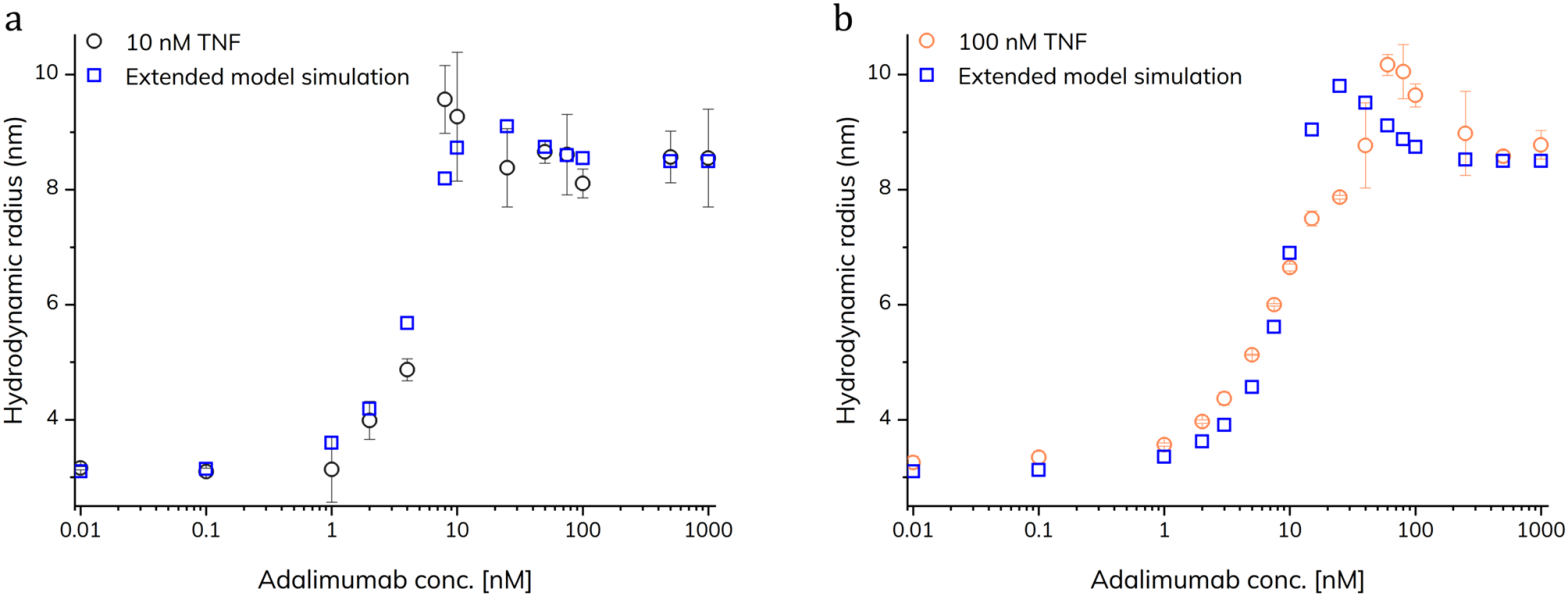
Extended model simulations of 10 (**a**) and 100 (**b**) nM TNF-α-alexa488 (open squares) as function of adalimumab concentration (0–1000 nM) compared to FIDA measurements in 67 mM phosphate buffer with 0.1% v/v HSA at pH 7.40 and 25 °C using pre-incubated samples (open circles, n = 3, error bars represent standard deviation).

The formation of a 2:2 (TNF-α:adalimumab) complex (${K}_{{\mathrm{d}}_{4}}$, ${K}_{{\mathrm{d}}_{5}}$ and ${K}_{{\mathrm{d}}_{6}}$) is expected to occur to a lesser extent than 1:1 (${K}_{{\mathrm{d}}_{1}}$), 2:1 (${K}_{{\mathrm{d}}_{2}}$), 1:2 (${K}_{{\mathrm{d}}_{3}}$) complexes as it involves multiple interactions leading to a substantial avidity effect. The apparent hydrodynamic radius measured is considered to be a weighted average of the hydrodynamic radii of T, TA, TA_2_ and (TA)_2_. Additional higher order structures, such as T_2_A_3_ and TA_3_, have also been reported^22,23^, but were not taken into account here because the used concentrations of TNF-α and adalimumab would most likely not favor these complexes. The binding scheme listed above can be simulated leading to estimates of the six *K*_d_’s, as shown in the Supplementary Information. It is assumed that ${K}_{{\mathrm{d}}_{1}},$ ${K}_{{\mathrm{d}}_{2}}$ and ${K}_{{\mathrm{d}}_{3}}$ as well as ${K}_{{\mathrm{d}}_{4}}$, ${K}_{{\mathrm{d}}_{5}}$ and ${K}_{{\mathrm{d}}_{6}}$ are identical. In our simulation, two data sets were used corresponding to 10 and 100 nM TNF-α (Fig. 3). The dispersion of TNF-α inside the capillary during FIDA analysis results in a dilution effect, thus the total TNF-α concentration was set to obtain the best possible fit (see Supplementary Information). The simulation parameters, i.e., hydrodynamic radii and *K*_d_ values in Table 1 were estimated according to the findings in Fig. 2 as well as to the ability of the model to fit the experimental data. The ${K}_{{\mathrm{d}}_{4}}$, ${K}_{{\mathrm{d}}_{5}}$ and ${K}_{{\mathrm{d}}_{6}}$ are avidity stabilized (two non-covalent bonds are formed in the binding event), thus the values are approximately an order of magnitude lower.

**Table 1:** Simulation parameters used to obtain the simulated curves at 10 and 100 nM TNF-α.

Complex size reported as hydrodynamic radius (nm)					Dissociation constant (nM)					
T	TA	TA_2_	(TA)_2_	T_2_A	${K}_{{\mathrm{d}}_{1}}$	${K}_{{\mathrm{d}}_{2}}$	${K}_{{\mathrm{d}}_{3}}$	${K}_{{\mathrm{d}}_{4}}$	${K}_{{\mathrm{d}}_{5}}$	${K}_{{\mathrm{d}}_{6}}$
3.1	7.0	8.5	12	7.5	3	3	3	0.2	0.2	0.2

The model and selected simulation parameters seem to explain and fit the experimental data well, including the hook shape of the binding curves (Fig. 3). However, at 100 nM TNF-α and 10–100 nM adalimumab (Fig. 3b) there is a minor discrepancy with the model. This may be due to higher order species not included in the model.

**Figure 4:**
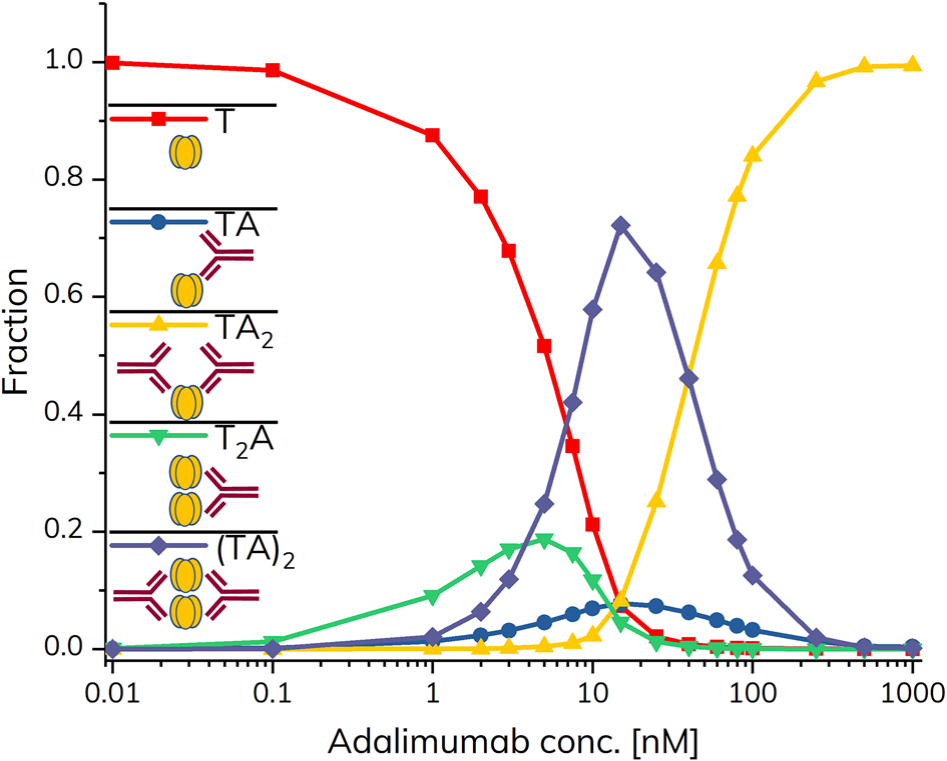
Simulated fractions of the various complexes between TNF-α and adalimumab as a function of adalimumab concentration corresponding to the experimental dataset at 100 nM TNF-α in the pre-incubated samples. Data points are based on the extended model simulations.

At low concentrations of adalimumab, TNF-α is the dominant species in solution whereas at high concentrations of adalimumab the dominant species is the TA_2_ complex. Interestingly, the amounts of the different species were heavily dependent on both the absolute and relative concentrations of TNF-α and adalimumab. Figure 4 shows how the fractions of the different species varies at a fixed TNF-α concentration of 100 nM in the pre-incubated samples. Under these conditions the large (TA)_2_ complex is the dominant species at intermediary adalimumab concentrations (~ 10 nM), as expected from the experimental data (Fig. 3b).

**Figure 5:**
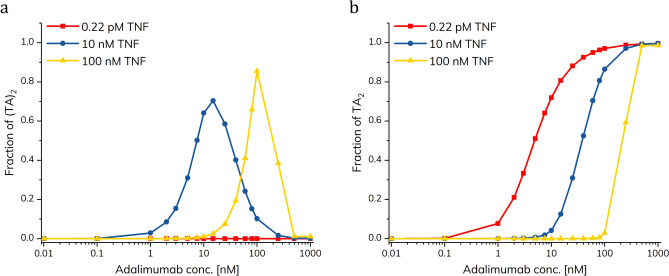
Simulated fractions of (TA)_2_ (**a**) and TA_2_ (**b**) as a function of adalimumab concentration. Simulations were performed using TNF-α concentrations of 100 nM, 10 nM, and 0.22 pM (healthy physiological condition).

Figure 5a shows how the fraction of (TA)_2_ is impacted by varying the TNF-α concentration. The (TA)_2_ complex is dominating at intermediary adalimumab concentrations (10 and 100 nM, respectively) when the TNF-α concentration is relatively high. However, when the simulation is performed using a TNF-α concentration corresponding to normal blood levels (0.22 pM), only a negligible fraction is bound in the (TA)_2_ complex. In case of inflammation, the TNF-α level may be significantly elevated thus resulting in increased formation of (TA)_2_. The formation of TA_2_ is shown at different TNF-α concentrations in Fig. 5b. As expected, the formation of this complex is shifted towards higher concentrations of adalimumab at higher concentrations of TNF-α.

The fractions of the complexes are strongly dependent on both the relative and absolute concentrations of TNF-α and adalimumab. The concentrations of complex-bound and free adalimumab as well as the ability to address binding stoichiometry provides important new information on the TNF-α–adalimumab system. The nature and the amounts of the complexes formed is not only of importance for characterizing the mode of action on a molecular level, but it may also provide information on possible adverse effects. For instance, it is speculated that some of the formed complexes are particularly prone to immunogenic responses in the patients^20,27,28^.

The fact that the TNF-α–adalimumab interactions results in several complexes being formed, also means that a simple 1:1 *K*_d_ does not provide a satisfactory description of the interaction and might be subject to large variations even upon small perturbations of the experimental conditions. Indeed, several techniques have been used to study the interaction between TNF-α and adalimumab. Krayukhina et al*.* used ITC (*K*_d_ = 2.2 nM^22^) at pH 7.4 and 25 °C. In comparison, surface based methods resulted in lower *K*_d_ values at similar pH and temperature: SPR (0.10 nM^21^, 0.18 nM^10^), BLI (0.005 nM^24^), and HP-SEC (0.24 nM^10^). Most of the *K*_d_ values reported in the literature assumes a 1:1 interaction, however, our results show that a strong avidity effect is needed to explain the sub-nanomolar *K*_d_ values observed with many surface binding technologies. The large variation reported in surface based *K*_d_ values could be a result of subtle differences in surface coverage and orientation of immobilized TNF-α/adalimumab at the surface, where small differences result in a substantial impact on the observed *K*_d_, since certain experimental conditions promote strong avidity effects. For binding systems composed of several active subunits (oligomers) it is therefore advisable to use orthogonal in-solution technologies to confirm or elucidate binding mechanisms and the associated *K*_d_ values.

### Characterization of TNF-α and adalimumab interactions in buffer (capillary mixed samples)

Characterization of biomolecular interactions using FIDA is often based on end-point measurements at equilibrium^14,15,29^, where binding kinetics are not probed. The experimental time frame can be precisely controlled in FIDA assays using an approach where the binding partners are mixed directly in the capillary^30^ (Fig. 1). This allows for an assessment of the binding kinetics. Based on the FIDA experiments, an apparent binding curve between TNF-α (100 nM) and adalimumab (0–1000 nM) was established using capillary mixing, where TNF-α-alexa488 and adalimumab were mixed inside the capillary during analysis, resulting in a reaction time less than 1.8 min (residence time inside the capillary).

**Figure 6:**
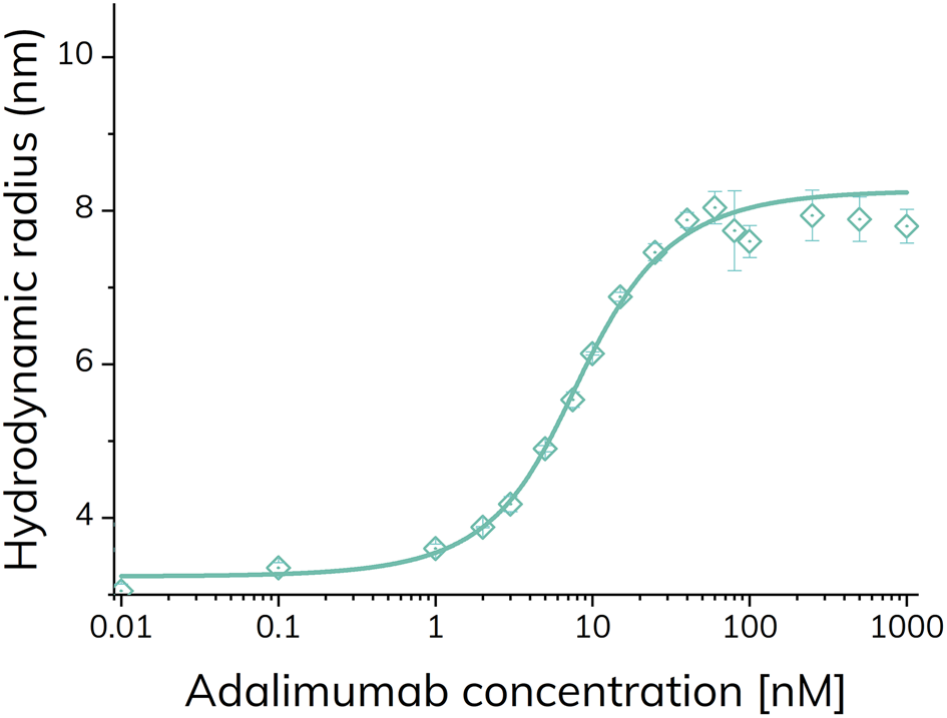
Apparent hydrodynamic radius of TNF-α-alexa488 (100 nM) as function of adalimumab concentration (0–1000 nM) determined by FIDA in 67 mM phosphate buffer with 0.1% v/v HSA at pH 7.40 and 25 °C (n = 3, error bars represent standard deviation) using capillary mixing (< 1.8 min). The solid turquoise line shows fitting to the excess indicator binding isotherm [Eq. (7)].

The apparent size of TNF-α increased with increasing adalimumab concentrations and reached a plateau value around 8.0 nm (Fig. 6). The 1:1 excess binding model (Eq. 7) resulted in an apparent dissociation constant (*K*_d_) of 1.75 nM and complex size of 8.3 nm (R^2^: 0.998). These findings were analogous to the results using the pre-incubated samples (Fig. 2), except that the higher order complex (presumably (TA)_2_) was not observed at equivalent concentrations (60–120 nM in Fig. 2). Apparently, higher order complexes did not form within the experimental time frame of 1.8 min. This suggests that the lower order complexes are formed relatively rapidly, whereas larger complexes and higher order stoichiometries require longer reaction times. The time-dependent and dynamic nature of these TNF-α adalimumab complexes have been observed previously in the literature using pre-incubated samples by SEC^21^.

### Characterization of TNF-α and adalimumab interactions in buffer (complex dissociation samples)

The dissociation kinetics of the formed TNF-α–adalimumab complexes was probed using the so-called complex dissociation mixing approach (Fig. 1). Here, the pre-incubated samples from Fig. 2 were surrounded by neat assay buffer inside the capillary, to induce complex dissociation.

**Figure 7:**
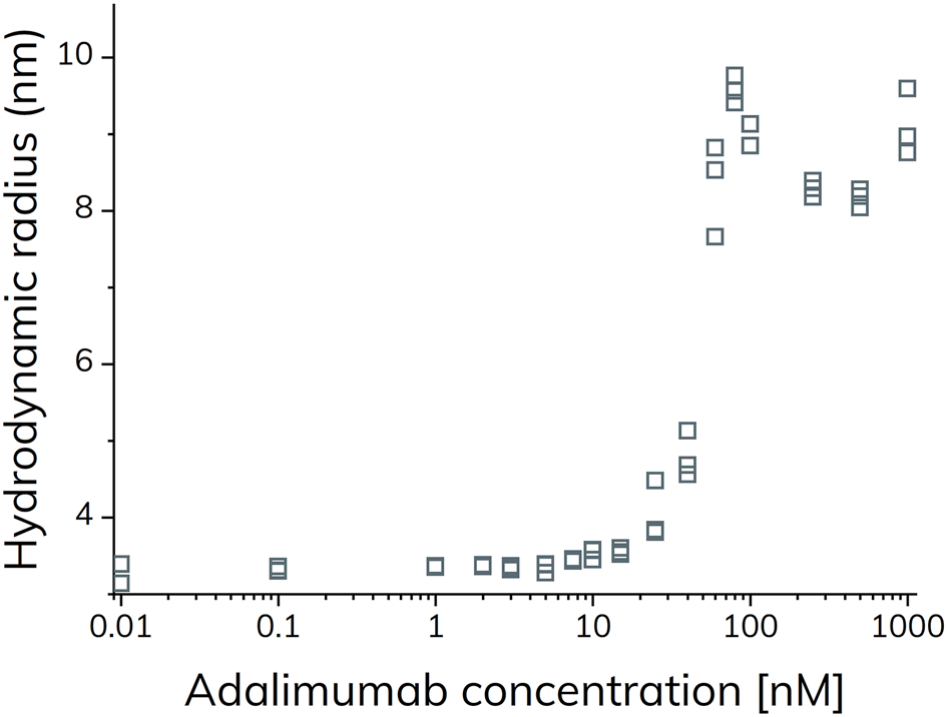
Apparent hydrodynamic radius of TNF-α-alexa488 (100 nM) as function of adalimumab concentration (0–1000 nM) determined by FIDA in 67 mM phosphate buffer with 0.1% v/v HSA at pH 7.40 and 25 °C (grey open squares) using complex dissociation.

Again, the apparent hydrodynamic radius of TNF-α increased with increasing adalimumab concentrations and reached a plateau value around 8.5–9.0 nm (Fig. 7). However, the apparent size change of TNF-α is shifted approximately an order of magnitude towards higher adalimumab concentrations as compared to Figs. 2 and 6, due to sample dilution in the capillary. The fact that no interaction is observed at low adalimumab concentration indicates a fast dissociation of the primary complexes (TA, T_2_A and TA_2_) relative to the mobilization time inside the capillary, whereas the avidity stabilized (TA)_2_ displays significantly slower dissociation kinetics as expected from SPR^10^ and hydrodynamic friction measurements^25^. According to Fig. 5a, the maximum amount of (TA)_2_ is observed at an adalimumab concentration around 100 nM, close to the sharp maximum at 70–80 nM adalimumab in Fig. 7. Interestingly, a second maximum is observed at the highest concentration of adalimumab (1000 nM) in Fig. 7, where the predominant species probably is the smaller TA_2_. Thus, the capillary dilution most likely shifts the equilibrium towards the avidity stabilized (TA)_2_ complex. Taken together, these results highlight the importance of considering the dilution effect of the analyte (adalimumab), for systems where the binding kinetics occurs on a timescale similar to the experiment.

### Characterization of TNF-α and adalimumab interactions in human plasma

The TNF-α–adalimumab interactions were further characterized in human plasma since this allows for the assessment of the complex formation under physiological relevant conditions. Binding between TNF-α (100 nM) and adalimumab (0–1000 nM) was investigated in human plasma from healthy donors diluted to 10 and 20% v/v plasma with assay buffer. These plasma concentrations were based on the expected steady-state concentrations of adalimumab in patients receiving adalimumab treatment with 40 mg every other week^19^, corresponding to 5.4 and 10.8 nM in 10 and 20% v/v plasma, respectively (see Supplementary Information). The dynamic range of the binding curve in Fig. 2, would therefore include relevant adalimumab plasma concentrations. The endogenous TNF-α concentration^31^ is several orders of magnitude below the indicator concentration of 100 nM, and thus not expected to interfere with the FIDA assay (see Supplementary Information).

The hydrodynamic radius of TNF-α, in absence of adalimumab (0 nM), was determined to be 3.3 ± 0.16 nm and 3.6 ± 0.32 nm in 10 and 20% v/v plasma, respectively, implying only very limited binding of TNF-α to plasma components when comparing with 3.20 ± 0.04 nm in neat assay buffer.

**Figure 8:**
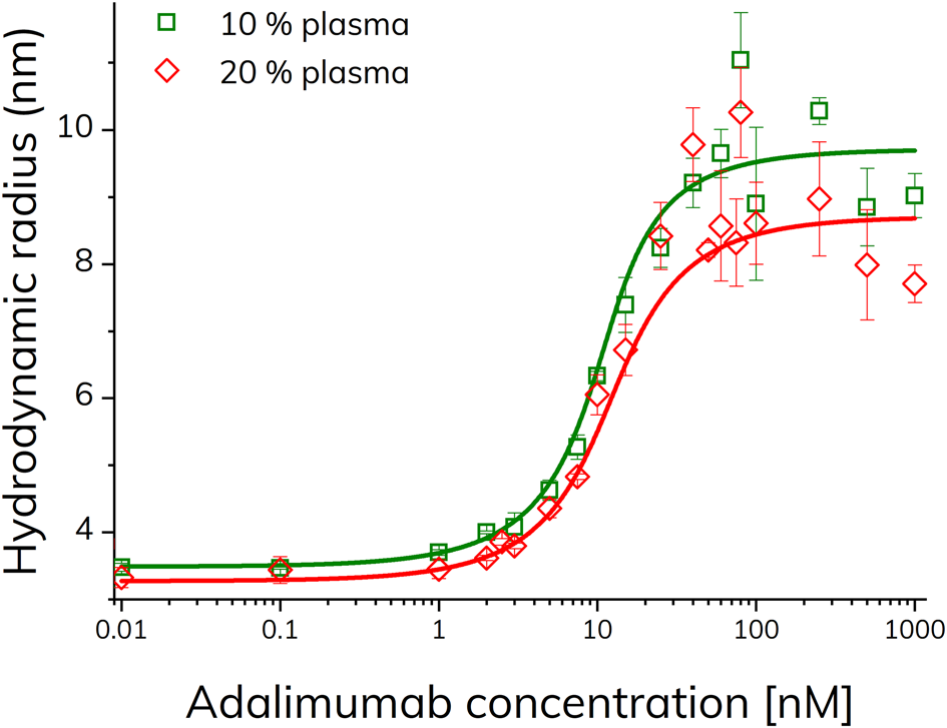
Apparent hydrodynamic radius of TNF-α-alexa488 (100 nM) as function of adalimumab concentration (0–1000 nM) in 10% v/v human plasma (green squares) and 20% v/v human plasma (red diamonds), determined by FIDA at 25 °C (n = 3, error bars represent standard deviation) using pre-incubated samples (> 10 min). The solid lines represent fitting to the excess indicator binding isotherm [Eq. (7)] with R^2^ of 0.987 (green) and 0.979 (red), respectively.

The binding curves (Fig. 8) showed an apparent increase in the size of TNF-α with increasing adalimumab concentrations as well as hook-shaped curves, similarly to the binding curve obtained in assay buffer (Fig. 2).

The 1:1 excess binding model [Eq. (7)] resulted in apparent *K*_d_ values of 1.01 and 1.73 nM, and complex sizes of 9.7 and 8.7 nm in 10 and 20% v/v plasma, respectively. Overall, these findings are in line with the apparent values in assay buffer (*K*_d_: 1.52 nM, complex size: 8.9 nm). The minor differences observed in complex size as function of adalimumab concentration emphasize that attention must be drawn to performing interaction and characterization assays under physiological relevant conditions, as they might differ from experiments performed in simple buffer systems.

## Conclusions

The present work demonstrates the application of FIDA assays for size-based and in-solution characterization of the interaction between TNF-α and adalimumab in both phosphate buffer and human plasma. We selectively measured the hydrodynamic radius of TNF-α in a titration series experiment with adalimumab, thereby determining the dissociation constant (*K*_d_) and complex sizes as well as confirming the oligomeric state of TNF-α. We found that the interaction involved generation of higher order complexes and substantial avidity effects. Therefore, the binding mechanism was modelled and simulated, demonstrating that the formation of higher order complexes highly depends on absolute and relative TNF-α and adalimumab concentrations. Furthermore, we performed an assessment of the binding kinetics, showing that smaller and lower order stoichiometries were rapidly formed whereas larger and higher order complexes required longer reaction time to form. Finally, the TNF-α–adalimumab interaction was measured in human plasma under physiological relevant conditions, implying similarities to the buffered model system, but also slight differences, thus stressing the importance of measuring in biological samples. The developed FIDA assays are applicable to measurement of both binding affinity and structural changes of biomolecular interactions under biorelevant conditions in a single assay format.

## Experimental

### Materials and chemicals

Recombinant human TNF-α (cat. no. ab155699) was procured from Abcam (Cambridge, United Kingdom). Adalimumab from Amgen Europe B.V. (Amgevita, Breda, Netherlands) was bought from Nomeco (Copenhagen, Denmark). The human plasma was received from Rigshospitalet (Copenhagen, Denmark). The biofluids were collected as anonymous human biological material (for waste), according to the required guidelines by the Danish Ethical Committee system and Data Protection Agency (Act on Research Ethics Review of Health Research Projects). All methods were carried out in accordance with the Danish Ethical committee system and Data Protection Agency, including any required informed consent.

Alexa Fluor 488 antibody labeling kit (cat. no. A20181) was purchased from Thermo Fisher (Waltham, MA, USA). Human serum albumin (HSA) was purchased from Sigma-Aldrich (St. Louis, Missouri, USA). Disodium hydrogen phosphate dihydrate, and sodium dihydrogen phosphate monohydrate were purchased from Merck (Darmstadt, Germany). Purified water (18.2 MΩ-cm at 25 °C) was obtained from an SG Ultraclear water purification system (SG Water, Barsbuttel, Germany).

#### Assay buffer

Phosphate buffer (pH 7.4; 67 mM) was prepared with purified water and filtered using a Q-max 0.45 µm nylon syringe filter (Frisenette, Knebel, Denmark) prior to use.

#### Fluorophore labelling of TNF-α

TNF-α was labelled with the Alexa Fluor 488 antibody labeling kit via tetrafluorophenyl (TFP) ester coupling chemistry, and subsequently purified, following the supplier’s instructions^32^. The TNF-α-alexa488 conjugate concentration was determined using a microvolume spectrophotometer (NanoDrop One, Thermo Fisher Scientific, Wilmington, Delaware, USA) applying the extinction coefficient of TNF-α (ε = 60,500 cm^−1^ M^−1^) at 280 nm^33^ assuming trimeric TNF-α in phosphate buffer (pH 7.4; 67 mM).

### Equipment

FIDA experiments were conducted using a Fida 1 instrument employing light-emitting-diode (LED) induced fluorescence detection with an excitation wavelength of 480 nm and emission wavelength > 515 nm (Fida Biosystems ApS, Copenhagen, Denmark). TDA experiments were performed using a Fida 1 instrument coupled to an intrinsic laser-induced-fluorescence (LIF) detector (ZETALIF evolution) with an excitation wavelength of 266 nm and emission wavelength of 300—760 nm (Picometrics, Labege, France).

PEG-coated capillaries with inner diameter 75 µm, outer diameter 375 µm, total length 100 cm, and length to detection window 84 cm (Fida Biosystems) were used for all experiments.

### Methods

#### FIDA and TDA procedures

For the TDA and FIDA experiments, the following protocol was applied. First, assay buffer (pH 7.4; 67 mM) was used for flushing and equilibrating the PEG-coated capillary at 1500 mbar for 300 s. Subsequently, the analyte solution was filled into the capillary at 1500 mbar for 45 s followed by the indicator sample at 50 mbar for 10 s. Finally, the indicator sample was mobilized to the fluorescence detector with analyte solution at 400 mbar for 180 s. The final mobilization step of the FIDA experiments conducted in plasma was set to 240 s at 400 mbar due to an increase in sample viscosity.

#### Hydrodynamic radius of adalimumab

The hydrodynamic radius of adalimumab (1 mg/mL) was measured in assay buffer by TDA employing intrinsic fluorescence detection.

#### Binding curves in buffer

Binding curves were generated using FIDA in assay buffer containing human serum albumin (0.1% v/v) as surface passivation agent. The TNF-α-alexa488 (indicator) concentration was fixed to 100 nM or 10 nM and titrated with adalimumab (analyte) in the concentration range 0–1000 nM. The different mixing schemes are described in Fig. 1. For the pre-incubated samples, TNF-α-alexa488 was pre-mixed with adalimumab in the sample vials for > 10 min to attain equilibrium before the FIDA measurements. The samples were analyzed in randomized order to confirm that no time effect on the results were in play.

#### Binding curves in plasma

Binding curves were generated using FIDA in 10 and 20% v/v human plasma diluted with assay buffer. The TNF-α-alexa488 concentration was fixed to 100 nM and titrated with adalimumab in the concentration range 0–1000 nM. TNF-α-alexa488 was pre-incubated with adalimumab in the sample vials for > 10 min to attain equilibrium before the FIDA measurements.

### Data analysis

The Taylorgrams were processed using the FIDA software suite, version 1.1 (Fida Biosystems ApS, Copenhagen, Denmark), in order to calculate the apparent hydrodynamic radius of TNF-α-alexa488 (indicator) at each adalimumab concentration (analyte). The Taylorgram fraction setting was set to 75% for all data points, and changes in sample viscosity were corrected according to a reference measurement at 25 °C, as previously described^14^.

A binding isotherm, assuming 1:1 binding and excess of indicator, was used for fitting the obtained FIDA measurements, i.e., apparent hydrodynamic radius of TNF-α-alexa488 as function of adalimumab concentration using the FIDA software^17^:

7 $$ R_{{{\text{app}}}} = \left( {\begin{array}{*{20}c} {\left( {R_{{{\text{IA}}}} } \right)^{ - 1} \cdot \left( {\frac{{\left( {C_{I} + C_{A} + K_{d} } \right) - \sqrt {\left( {C_{I} + C_{A} + K_{d} } \right)^{2} - 4 \cdot C_{A} \cdot C_{I} } )}}{{2 \cdot C_{I} }}} \right)} \\ { + \left( {R_{{\text{I}}} } \right)^{ - 1} \cdot \left( {\frac{{\left( {C_{I} - C_{A} - K_{d} } \right) + \sqrt {\left( {C_{I} + C_{A} + K_{d} } \right)^{2} - 4 \cdot C_{A} \cdot C_{I} } )}}{{2 \cdot C_{I} }}} \right)} \\ \end{array} } \right)^{ - 1} $$

where *R*_app_, *R*_I_*,* and *R*_IA_ are the apparent, indicator and complex hydrodynamic radii, respectively, *K*_d_ is the dissociation constant, and *C*_I_ and *C*_A_ are the formal concentrations of the indicator and analyte, respectively.

An extended binding model for multiple and higher order stoichiometries is derived in the results and discussion paragraph as well as in the Supplementary Information.

## Supplementary information

Supplementary information.

## Data Availability

The datasets generated and analyzed during the current study are available from the corresponding author on request.
