# Supplementary material for: Size-based characterization of adalimumab and TNF-α interactions using flow induced dispersion analysis: assessment of avidity-stabilized multiple bound species
Source: Sci Rep. 2021 Feb 26;11:4754. doi: 10.1038/s41598-021-84113-z (PMC7910425; doi:10.1038/s41598-021-84113-z)
Supplement: Supplementary file 1 — Supplementary information. [file 41598_2021_84113_MOESM1_ESM.docx]

**Supplementary Information**

# Size-based characterization of adalimumab and TNF-α interactions using Flow Induced Dispersion Analysis: Assessment of Avidity-stabilized Multiple Bound Species

Morten E. Pedersen^†,a,b^, Ragna M.S. Haegebaert^†,b^, Jesper Østergaard^b^, and Henrik Jensen^a,b^

*^a^Fida Biosystems ApS, Fruebjergvej 3, 2100 Copenhagen Ø, Denmark.*

*^b^Department of Pharmacy, University of Copenhagen, Universitetsparken 2, 2100 Copenhagen O, Denmark.*

^†^ *These authors contributed equally*

**Raw data for establising the binding curve**

In figure S1 an overlay of normalized raw data used to obtain Figure 2 is shown. The figure clearly shows the peak broardening in response to binding. In order to facilitate comparison, the data were normalized to the retention time as described in the literature^S1^.


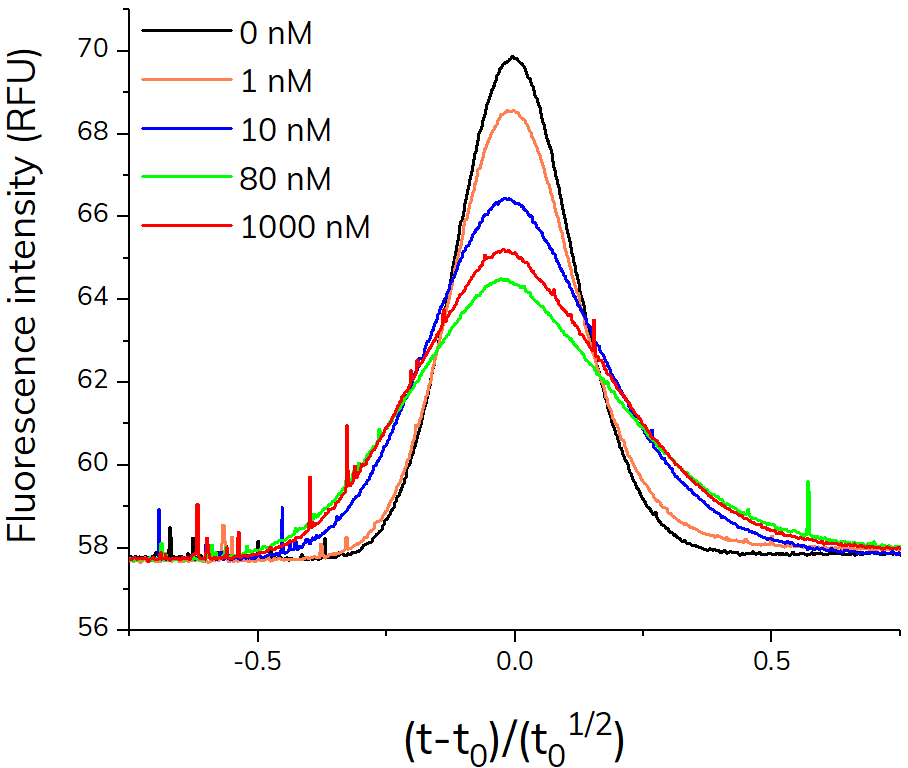


*Figure S1: Normalized raw data (i.e., Taylorgrams) of TNF-α-alexa488 in presence of increasing concentrations of adalimumab used to obtain the binding curve in Figure 2 in the main manuscript.*

**Extended binding model**

In this work, we assume the presence of only 1:1, 1:2, 2:1 and 2:2 (TNF-α - adalimumab) binding stoichiometries described by the following equilibria:

$\mathrm{TA}\rightleftharpoons T+A$ (S1)

$T_{2}A\rightleftharpoons TA+T$ (S2)

$TA_{2}\rightleftharpoons\mathrm{TA}+A$ (S3)

$(T{A)}_{2}\rightleftharpoons\mathrm{TA}+\mathrm{TA}$ (S4)

$(T{A)}_{2}{\rightleftharpoons T}_{2}A+A$ (S5)

$(T{A)}_{2}\rightleftharpoons TA_{2}+T$ (S6)

where T is the indicator (i.e. TNF-α in the present work) and A is the analyte (adalimumab in the present work). TA, TA_2_, T_2_A and (TA)_2_ are the 1:1, 1:2, 2:1 and 2:2 complexes formed from TNF-α and adalimumab, respectively. These complexes have previously been described in the literature^S2-S3^.

The dissociation constants (*K*_d_) corresponding to S1-S6 can be written as:

$K_{d_{1}}=\frac{\left[ T \right] \cdot[A]}{[\mathrm{TA}]}$ (S7)

$K_{d_{2}}=\frac{[\mathrm{TA}]\cdot[T]}{[T_{2}A]}$ (S8)

$K_{d_{3}}=\frac{\left[ \mathrm{TA} \right] \cdot[A]}{[TA_{2}]}$ (S9)

$K_{d_{4}}=\frac{\left[ \mathrm{TA} \right]^{2}}{[{(TA)}_{2}]}$ (S10)

$K_{d_{5}}=\frac{[T_{2}A] \cdot[A]}{[{(TA)}_{2}]}$ (S11)

$K_{d_{6}}=\frac{[TA_{2}] \cdot[T]}{[{(TA)}_{2}]}$ (S12)

where [T], [A], [TA], [TA_2_], $[T_{2}A]$ and [(TA)_2_] are the actual TNF-α, adalimumab, 1:1 complex, 1:2 complex, 2:1 complex and 2:2 complex concentrations, respectively.

The formal concentration of T (*C_T_*) can be written as:

$C_{T}=\left[ T \right]+\left[ \mathrm{TA} \right]+\left[ TA_{2} \right]+2\cdot[\left( \mathrm{TA} \right)_{2}]$ + 2 · [$T_{2}A]$ (S13)

The formal concentration of A (*C*_A_) can be written as:

$C_{A}=\left[ A \right]+\left[ \mathrm{TA} \right]+2\cdot\left[ TA_{2} \right]+2\cdot[\left( \mathrm{TA} \right)_{2}]$ + [$T_{2}A]$ (S14)

From which the actual adalimumab concentration is obtained as

$\left[ A \right]=C_{A}-\left( \left[ \mathrm{TA} \right]+2\cdot\left[ TA_{2} \right]+2\cdot[\left( \mathrm{TA} \right)_{2}] + [T_{2}A] \right)$ (S15)

The actual concentrations of the T containing species can be expressed according to the concentration of T by employing equation S1-S3:

$\left[ \mathrm{TA} \right]=\frac{\left[ T \right] \cdot[A]}{K_{d_{1}}}$ (S16)

$\left[ TA_{2} \right]=\frac{\left[ T \right] \cdot\left[ A \right]^{2}}{K_{d_{1}}\cdot K_{d_{3}}}$ (S17)

[$T_{2}A]= \frac{\left[ A \right] \cdot\left[ T \right]^{2}}{K_{d_{1}}\cdot K_{d_{2}}}$ (S18)

The concentration of $\left( \mathrm{TA} \right)_{2}$ is determined by S4 – S6. In order to take into account all three dissociation pathways the equilibria are summed up to give the following expression for the total dissociation:

$K_{d_{4}}\cdot K_{d_{5}}\cdot K_{d_{6}}= \frac{[T_{2}A] \cdot[A]}{[{(TA)}_{2}]}\cdot\frac{\left[ \mathrm{TA} \right]^{2}}{[{(TA)}_{2}]}\cdot\frac{[TA_{2}] \cdot[T]}{[{(TA)}_{2}]}$ (S19)

${[{(TA)}_{2}]}^{3}=\frac{\left[ T \right]^{2}\cdot\left[ A \right]^{2}}{{K_{d_{1}}}^{2}\cdot K_{d_{4}}\cdot K_{d_{5}}}\cdot\frac{\left[ A \right]^{2} \cdot\left[ T \right]^{2}}{K_{d_{1}}\cdot K_{d_{2}}\cdot K_{d_{6}}}\cdot\frac{\left[ T \right]^{2}\cdot\left[ A \right]^{2}}{K_{d_{1}}\cdot K_{d_{3}}}$ (S20)

$[{(TA)}_{2}]$ = $\frac{\left[ T \right]^{2}\cdot\left[ A \right]^{2}}{\sqrt[3]{{K_{d_{1}}}^{4}\cdot K_{d_{4}}\cdot K_{d_{5}}\cdot{K_{d_{6}}\cdot K_{d_{3}}\cdot K}_{d_{2}}}}$ (S21)

According to S1-S6 the complete dissociation of (TA)_2_ can follow three distinct pathways all resulting in the complete dissociation into T and A. The thermodynamic consequence is that:

${K_{d_{1}}}^{2}\cdot K_{d_{4}}=K_{d_{1}}\cdot{K_{d_{2}}\cdot K_{d_{5}}=K_{d_{1}}\cdot K}_{d_{3}}\cdot K_{d_{6}}$ (S22)

Further, based on the chemical composition of the formed complexes we shall simplify the model assuming that $K_{d_{4}}= K_{d_{5}}=K_{d_{6}}$ and $K_{d_{1}}=K_{d_{2}}= K_{d_{3}}$.

Under this assumption S21 reduces to:

$[{(TA)}_{2}]$ =$\left[ T \right]^{2}\cdot$ $\frac{\left[ A \right]^{2}}{{K_{d_{1}}}^{2}\cdot K_{d_{4}}}$ (S23)

Substituting S16-S18 and S23 into S13 yields:

$C_{T}=\left[ T \right]+\frac{\left[ T \right] \cdot[A]}{K_{d_{1}}}+\frac{\left[ T \right] \cdot\left[ A \right]^{2}}{K_{d_{1}}\cdot K_{d_{3}}}+2\cdot\frac{\left[ T \right]^{2}\cdot\left[ A \right]^{2}}{K_{d_{1}}^{2}\cdot K_{d_{4}}}+2\cdot\frac{\left[ A \right] \cdot\left[ T \right]^{2}}{K_{d_{1}}\cdot K_{d_{2}}}$ (S24)

Rearranging eq S21 results to the following second order equation provides:

${\left[ T \right]^{2}\left( 2\cdot\frac{\left[ A \right]^{2}}{K_{d_{1}}^{2}\cdot K_{d_{4}}}+2\cdot\frac{\left[ A \right]}{K_{d_{1}}\cdot K_{d_{2}}} \right)+\left[ T \right]\left( 1+\frac{[A]}{K_{d_{1}}}+\frac{\left[ A \right]^{2}}{K_{d_{1}}\cdot K_{d_{3}}} \right)-C}_{T}=0$ (S25)

Defining

$A_{T}=2\cdot\frac{\left[ A \right]^{2}}{K_{d_{1}}^{2}\cdot K_{d_{4}}}+2\cdot\frac{\left[ A \right]}{K_{d_{1}}\cdot K_{d_{2}}}$ (S26)

$B_{T}=1+\frac{[A]}{K_{d_{1}}}+\frac{\left[ A \right]^{2}}{K_{d_{1}}\cdot K_{d_{3}}}$ (S27)

equation S22 can then be written as:

${\left[ T \right]^{2}{\cdot A}_{T}+\left[ T \right]\cdot B_{T}-C}_{T}=0$ (S28)

Equation S25 has the following chemically meaningful closed form solution:

$\left[ T \right]=\frac{-B_{T} +\sqrt{B_{T}^{2} + 4\cdot A_{T}{\cdot C}_{T}}}{2\cdot A_{T}}$ (S29)

Following a similar strategy for [TA],$C_{T}$ can be linked to [TA] according to:

${\left[ \mathrm{TA} \right]^{2}\left( 2\cdot\sqrt[3]{\frac{{K_{d_{1}}}^{2}}{K_{d_{2}}\cdot K_{d_{3}}\cdot K_{d_{4}}\cdot K_{d_{5}}K_{d_{6}}}}+2\cdot\frac{K_{d_{1}}}{\left[ A \right]\cdot K_{d_{2}}} \right)+\left[ \mathrm{TA} \right]\left( 1+\frac{K_{d_{1}}}{[A]}+\frac{\left[ A \right]}{K_{d_{3}}} \right)-C}_{T}=0$ (S30)

Assuming as before that $K_{d_{4}}= K_{d_{5}}=K_{d_{6}}$ and $K_{d_{1}}=K_{d_{2}}=K_{d_{3}}$ equation S30 reduces to:

${\left[ \mathrm{TA} \right]^{2}\left( 2\cdot\frac{1}{K_{d_{4}}}+2\cdot\frac{K_{d_{1}}}{\left[ A \right]\cdot K_{d_{2}}} \right)+\left[ \mathrm{TA} \right]\left( 1+\frac{K_{d_{1}}}{[A]}+\frac{\left[ A \right]}{K_{d_{3}}} \right)-C}_{T}=0$ (S31)

Defining

$A_{\mathrm{TA}} = 2\cdot\frac{1}{K_{d_{4}}}+2\cdot\frac{K_{d_{1}}}{\left[ A \right]\cdot K_{d_{2}}}$ (S32)

$B_{\mathrm{TA}}=1+\frac{K_{d_{1}}}{[A]}+\frac{\left[ A \right]}{K_{d_{3}}}$ (S33)

Equation S31 can be written:

${\left[ \mathrm{TA} \right]^{2}{\cdot A}_{\mathrm{TA}}+\left[ \mathrm{TA} \right]\cdot B_{\mathrm{TA}}-C}_{T}=0$ (S34)

Equation S34 has the following physically meaningful closed form solution:

$\left[ \mathrm{TA} \right]=\frac{-B_{\mathrm{TA}} +\sqrt{B_{\mathrm{TA}}^{2} + 4\cdot A_{\mathrm{TA}}{\cdot C}_{T}}}{2\cdot A_{\mathrm{TA}}}$ (S35)

The actual concentrations of $\left[ TA_{2} \right]$and [$T_{2}A]$ are found from equations S8 and S9, and $[\left( \mathrm{TA} \right)_{2}]$ is finally obtained from S13.

In cases where the concentration of T is very low compared to A, it is a good approximation to use $C_{A}$ in place of [A]. This is, however, not the case for the current set of data. The equation system is therefore simulated for a given set of *C*_T_ and $K_{d_{1}}-K_{d_{6}}$ by using guesses of [A] to calculate concentrations of all the remaining species as listed above. An apparent concentration of *C*_T_ is used in order to take into account dilution of T in the capillary. The “actual” [A] is calculated from equation S15 and finally the rooth mean square (rms) difference between “guessed” and “actual” [A] is obtained. For the present system, an evolutionary protocol is used and implemented in Excel using the solver package and the evolutionary solver tool. The optimization criterium is based on the sum of the rms difference of all the measured concentrations. Excel sheets are available upon request.

The actual measurement is an apparent hydrodynamic radius (*R*_h_) of the fluorescently labeled TNF-α. The apparent hydrodynamic radius is linked to an apparent diffusivity. For a simple 1:1 binding, it has previously been shown that the inverse of the measured apparent *R*_h_ can be modeled as a weighted average of the inverse radius of the fraction bound and unbound^S4^. A similar model is used in the present case, where the relative fractions of the different species is calculated from the concentrations obtained as described above. The relative fractions of T, TA, TA_2_, $T_{2}A$ and (TA)_2_ are termed $x_{T}$ , $x_{\mathrm{TA}}, x_{\mathrm{TA}_{2}}, x_{T_{2}A}\mathrm{and}x_{\left( \mathrm{TA} \right)_{2}} ,$respectively.

$x_{T}=\frac{[T]}{C_{T}}$ (S36)

$x_{\mathrm{TA}}=\frac{[\mathrm{TA}]}{C_{T}}$ (S37)

$x_{T_{2}A}=\frac{[T_{2}A]}{C_{T}}$ (S38)

$x_{\mathrm{TA}_{2}}=\frac{[\mathrm{TA}_{2}]}{C_{T}}$ (S39)

$x_{\left( \mathrm{TA} \right)_{2}}=\frac{[\left( \mathrm{TA} \right)_{2}]}{C_{T}}$ (S40)

The measured apparent hydrodynamic radius (*R*_h,app_) is then obtained as:

$R_{h,app}= \left( \frac{1}{R_{T}}{\cdot x}_{T}+\frac{1}{R_{\mathrm{TA}}}{\cdot x}_{\mathrm{TA}}+\frac{1}{R_{T_{2}A}}{\cdot x}_{T_{2}A}+\frac{1}{R_{\mathrm{TA}_{2}}}\cdot x_{\mathrm{TA}_{2}}+\frac{1}{R_{\left( \mathrm{TA} \right)_{2}}}{\cdot x}_{\left( \mathrm{TA} \right)_{2}} \right)^{-1}$ (S41)

where $R_{T}, R_{\mathrm{TA}}, R_{T_{2}A}, R_{\mathrm{TA}_{2}} \mathrm{and}R_{\left( \mathrm{TA} \right)_{2}}$ are the hydrodynamic radii of T, TA, T_2_A TA_2_ and (TA)_2_, respectively.

The simulation is performed for different *C*_T_ values as well as for different *K*_d_ values and hydrodynamic radii in order to arrive at a simulation which represents the measured data well. The input parameters are manually optimised until an optimal fit to the data. The optimal *K*_d_´s are listed in the main manuscript. *C*_T_ values of 12 nM and 5 nM were used to fit experimental dataset (figure 2A, 2B and 3) corresponding to 100 nM and 10 nM TNF-α respectively. The lower *C*_T_ takes into account capillary dilution effects. The simulation in figure 4 was performed using CT values of 0.22 pM, 10 nM and 100 nM, respectively.

**Steady-state concentration of adalimumab in RA patients**

Rheumatoid arthritis patients administered 40 mg every other week have a mean steady state serum concentration of 8 µg/mL^S5^. This corresponds to 54 nM, using a molecular weight of 148 kDa for adalimumab^S5^. Thus, the expected adalimumab concentration is 5.4 and 10.8 nM in 10 and 20 % v/v plasma, respectively.

**Endogenous TNF-α level**

The endogenous TNF-α serum level for healthy individuals has been reported as 11.2 ± 7.31 pg/mL^S6^, corresponding to 0.22 ± 0.14 pM in 100 % serum using a molecular weight of 52 kDa for TNF-α. Thus, the concentration is 0.022 and 0.044 pM in 10 and 20 % v/v plasma, respectively.

**References**

S1. Chamieh, J.; Merdassi, H.; Rossi, J. C.; Jannin, V.; Demarne, F.; Cottet, H. Size Characterization of Lipid-Based Self-Emulsifying Pharmaceutical Excipients during Lipolysis Using Taylor Dispersion Analysis with Fluorescence Detection. *Int. J. Pharm*. **2018**, *537* (1–2), 94–101.

S2. Krayukhina, E. *et al.* Analytical ultracentrifugation with fluorescence detection system reveals differences in complex formation between recombinant human TNF and different biological TNF antagonists in various environments. *MAbs* **9**, 664–679 (2017).

S3. Tran, B. N. *et al.* Higher order structures of Adalimumab, Infliximab and their complexes with TNFα revealed by electron microscopy. *Protein Sci.* **26**, 2392–2398 (2017).

S4. Pedersen, M. E., Østergaard, J. & Jensen, H. In-Solution IgG Titer Determination in Fermentation Broth Using Affibodies and Flow-Induced Dispersion Analysis. *ACS Omega* **5**, 10519–10524 (2020).

S5. Abbvie. Humira Product monograph. 174 (2019).

S6. Arican, O., Aral, M., Sasmaz, S. & Ciragil, P. Serum levels of TNF-α, IFN-γ, IL-6, IL-8, IL-12, IL-17, and IL-18 in patients with active psoriasis and correlation with disease severity. *Mediators Inflamm.* **5**, 273–279 (2005).
